# Supplementary material for: Heat adaptation of phage T7 under an extended genetic code
Source: Virus Evol. 2021 Dec 1;7(2):veab100. doi: 10.1093/ve/veab100 (PMC8923235; doi:10.1093/ve/veab100)
Supplement: veab100_Supp [file veab100_supp.zip › Sequencing_Analysis.zip!/Sequencing_Analysis/S2/evidence/JC_68.html]

BRESEQ :: Evidence


| New junction evidence | | | | | | | | | | | |
| --- | --- | --- | --- | --- | --- | --- | --- | --- | --- | --- | --- |
|  | | seq id | position | reads (cov) | reads (cov) | score | skew | freq | annotation | gene | product |
| \* | ? | NC\_001604.1 | = 16604 | 3197 (0.600) | 1690 (0.320) | 335/590 | 1.7 | 34.5% | intergenic (–/–) | *–/–* | –/– |
|  | ? | NC\_001604.1 | 17028 = | 3275 (0.630) |  |  |  |  | intergenic (–/–) | *–/–* | –/– |

Only 100 of 1981 total aligned reads displayed.  

`GGCATGGGTACATGATGAAATCCAAGTAGGCTGCCGTACCGAAGAGATTGCTCAGGTGGTCATTGAGACCGCACAAGAAGCGATGCGCTGGGTTGGAGACCACTGGAACTTCCGGTGTCTTCTGGATACCGAAGGTAAGATGGGTCCTAATTGGGCGATTTGCCACTGATACAGGAGGCTACTCATGAACGAAAGACACTTAACAGGTGCTGCTTCTGAAATGCTAGTAGCCTACAAATTTACCAAAGCTGGGTACACTGTCTATTACCCTATGCTGACTCAGAGTAAAGAGGACTTGGTTGTATG‑‑‑‑‑‑‑‑‑‑‑‑‑‑‑‑‑‑‑‑‑‑‑‑‑‑‑‑‑‑‑‑‑‑‑‑‑‑‑‑‑‑‑‑‑‑‑‑‑‑‑‑‑‑‑‑‑‑‑‑‑‑‑‑‑‑‑‑‑‑‑‑‑‑‑‑‑‑‑‑‑‑‑‑‑‑‑‑‑‑‑‑‑‑‑‑‑‑‑‑‑‑‑‑‑‑‑‑‑‑‑‑‑‑‑‑‑‑‑‑‑‑‑‑‑‑‑‑‑‑‑‑‑‑‑‑‑‑‑‑‑‑‑‑‑‑‑‑‑‑‑‑‑‑‑‑‑‑‑‑‑‑‑‑‑‑‑‑‑‑‑‑‑‑‑‑‑‑‑‑‑‑‑‑‑‑‑‑‑‑‑‑‑‑‑‑‑‑‑‑‑‑‑‑‑‑‑‑‑‑‑‑‑‑‑‑‑‑‑‑‑‑‑‑‑‑‑‑‑‑‑‑‑‑‑‑‑‑‑‑‑‑‑‑‑‑‑‑‑‑‑‑‑‑‑‑‑‑‑‑‑‑‑‑‑‑‑‑‑‑‑‑‑‑‑‑‑‑‑‑‑‑‑‑‑‑‑‑‑‑‑‑‑‑‑‑‑‑‑‑  >  NC_001604.1/16299‑16604`  
`‑‑‑‑‑‑‑‑‑‑‑‑‑‑‑‑‑‑‑‑‑‑‑‑‑‑‑‑‑‑‑‑‑‑‑‑‑‑‑‑‑‑‑‑‑‑‑‑‑‑‑‑‑‑‑‑‑‑‑‑‑‑‑‑‑‑‑‑‑‑‑‑‑‑‑‑‑‑‑‑‑‑‑‑‑‑‑‑‑‑‑‑‑‑‑‑‑‑‑‑‑‑‑‑‑‑‑‑‑‑‑‑‑‑‑‑‑‑‑‑‑‑‑‑‑‑‑‑‑‑‑‑‑‑‑‑‑‑‑‑‑‑‑‑‑‑‑‑‑‑‑‑‑‑‑‑‑‑‑‑‑‑‑‑‑‑‑‑‑‑‑‑‑‑‑‑‑‑‑‑‑‑‑‑‑‑‑‑‑‑‑‑‑‑‑‑‑‑‑‑‑‑‑‑‑‑‑‑‑‑‑‑‑‑‑‑‑‑‑‑‑‑‑‑‑‑‑‑‑‑‑‑‑‑‑‑‑‑‑‑‑‑‑‑‑‑‑‑‑‑‑‑‑‑‑‑‑‑‑‑‑‑‑‑‑‑‑‑‑‑‑‑‑‑‑‑‑‑‑‑‑‑‑‑‑‑‑‑‑‑‑‑‑‑‑‑‑‑‑‑‑gtatgGAAGGATTGATGACATTCGTAGTACGTACATCATTTCGTGAGGCCATTAAGGACATGCACGAAGAGTATGCAGATAAGGACTCTTTCAAACAATCTCCTGCAACAGTACGGGAGGTGTTCTGATGTCTGACTACCTGAAAGTGCTGCAAGCAATCAAAAGTTGCCCTAAGACTTTCCAGTCCAACTATGTACGGAACAATGCGAGCCTCGTAGCGGAGGCCGCTTCCCGTGGTCACATCTCGTGCCTGACTACTAGTGGACGTAACGGTGGCGCTTGGGAAATCACTGCTTCCGGTACTC  >  NC_001604.1/17028‑17327`  

                                                                                                                                                                                                                                                                                                                                                                                                                                                                                                                                                                                                                                
 `GCATGGGTACATGATGAAATCCAAGTAGGCTGCCGTACCGAAGAGATTGCTCAGGTGGTCATTGAGACCGCACAAGAAGCGATGCGCTGGGTTGGAGACCACTGGAACTTCCGGTGTCTTCTGGATACCGAAGGTAAGATGGGTCCTAATTGGGCGATTTGCCACTGATACAGGAGGCTACTCATGAACGAAAGACACTTAACAGGTGCTGCTTCTGAAATGCTAGTAGCCTACAAATTTACCAAAGCTGGGTACACTGTCTATTACCCTATGCTGACTCAGAGTAAAGAGGACTTGGTTG                                                                                                                                                                                                                                                                                                                  >  1:603828/1‑301`  
 `GCATGGGTACATGATGAAATCCAAGTAGGCTGCCGTACCGAAGAGATTGCTCAGGTGGTCATTGAGACCGCACAAGAAGCGATGCGCTGGGTTGGAGACCACTGGAACTTCCGGTGTCTTCTGGATACCGAAGGTAAGATGGGTCCTAATTGGGCGATTTGCCACTGATACAGGAGGCTACTCATGAACGAAAGACACTTAACAGGTGCTGCTTCTGAAATGCTAGTAGCATACAAATTTACCAAAGCTGGGTACACTGTCTATTACCCTATGCTGACTCAGAGTAAAGAGGACTTGGTTG                                                                                                                                                                                                                                                                                                                  >  1:246936/1‑301`  
    `TGGGTACATGATGAAATCCAAGTAGGCTGCCGTACCGAAGAGATTGCTCAGGTGGTCATTGAGACCGCACAAGAAGCGATGCGCTGGGTTGGAGACCACTGGAACTTCCGGTGTCTTCTGGATACCGAAGGTAAGATGGGTCCTAATTGGGCGATTTGCCACTGATACAGGAGGCTACTCATGAACGAAAGACACTTAGCAGGTGCTGCTTCTGAAATGCTAGTAGCCTACAAATTTACCAAAGCTGGGTACACTGTCTATTACCCTATGCTGACTCAGAGTAAAGCGGACTTGGTTGTAT                                                                                                                                                                                                                                                                                                               >  1:215411/1‑301`  
    `TGGGTACATGATGAAATCCAAGTAGGCTGCCGTACCGAAGAGATTGCTCAGGTGGTCATTGAGACCGCACAAGAAGCGATGCGCTGGGTTGGAGACCACTGGAACTTCCGGTGTCTTCTGGATACCGAAGGTAAGATGGGTCCTAATTGGGCGATTTGCCACTGATACAGGAGGCTACTCAAGAACGAAAGACACTTAACAGGTGCTGCTTCTGAAATGCTAGTAGCCTACAAATTTACCAAAGCTGGGTACACTGTCTATTACCCTATGCTGACTCAGAGTAAAGAGGACTTGGTTGTAT                                                                                                                                                                                                                                                                                                               <  1:366347/301‑1`  
     `tGGTACATGATGAAATCCAAGTAGGCTGCCGTACCGAAGAGATTGCTCAGGTGGTCATTGAGACCGCACAAGAAGCGATGCGCTGGGTTGGAGACCACTGGAACTTCCGGTGTCTTCTGGATACCGAAGGTAAGATGGGTCCTAATTGGGCGATTTGCCACTGATACAGGAGGCTACTCATGAACGAAAGACACTTAACAGGTGCTGCTTCTGAAATGCTAGTAGCCTACAAATTTACCAAAGCTGGGTACACTGTCTATTACCCTATGCTGACTCAGAGTAAAGAGGACTTGGTTGTATG                                                                                                                                                                                                                                                                                                              <  1:637487/300‑1`  
     `agagACAGGATGAAATCCAAGTAGGCTGCCGTACCGAAGAGATTGCTCAGGTGGTCATTGAGACCGCACAAGAAGCGATGCGCTGGGTTGGAGACCACTGGAACTTCCGGTGTCTTCTGGATACCGAAGGTAAGATGGGTCCTAATTGGGCGATTTGCCACTGATACAGGAGGCTACTCATGAACGAAAGACACTTAACAGGTGCTGCTTCTGAAATGCTAGTAGCCTACAAATTTACCAAAGCTGGGTACACTGTCTATTACCCTATGCTGACTCAGAGTAAAGAGGACTTGGTTGTATG                                                                                                                                                                                                                                                                                                              <  1:284752/297‑1`  
     `GGGTACATGATGAAATCCAAGTAGGCTGCCGTACCGAAGAGATTGCTCAGGTGGTCATTGAGACCGCACAAGAAGCGATGCGCTGGGTTGGAGACCACTGGAACTTCCGGTGTCTTCTGGATACCGAAGGTAAGATGGGTCCTAATTGGGCGATTTGCCACTGATACAGGAGGCTACTCATGAACGAAAGACACTTAACAGGTGCTGCTTCTGAAATGCTAGTAGCCTACAAATTTACCAAAGCTGGGTACACTGTCTATTACCCTATGCTGACTCAGAGTAAAGAGGACTTGGTTGTATG                                                                                                                                                                                                                                                                                                              >  1:491936/1‑301`  
     `GGGTACATGATGAAATCCAAGTAGGCTGCCGTACCGAAGAGATTGCTCAGGTGGTCATTGAGACCGCACAAGAAGCGATGCGCTGGGTTGGAGACCACTGGAACTTCCGGTGTCTTCTGGATACCGAAGGTAAGATGGGTCCTAATTGGGCGATTTGCCACTGATACAGGAGGCTACTCATGAACGAAAGACACTTAACAGGTGCTGCTTCTGAAATGCTAGTAGCCTACAAATTTACCAAAGCTGGGTACACTGTATATTACCCTATGCTGACTCAGAGTAAAGAGGACTTGGTTGTATG                                                                                                                                                                                                                                                                                                              >  1:359586/1‑301`  
      `gtTACATGATGAAATCCAAGTAGGCTGCCGTACCGAAGAGATTGCTCAGGTGGTCATTGAGACCGCACAAGAAGCGATGCGCTGGGTTGGAGACCACTGGAACTTCCGGCGTCTTCTGGATACCGAAGGTAAGATGGGTCCTAATTGGGCGATTTGCCACTGATACAGGAGGCTACTCATGAACGAAAGACACTTAACAGGTGCTGCTTCTGAAATGCTAGTAGCCTACAAATTTACCAAAGCTGGGTACACTGTCTATTACCCTATGCTGACTCAGAGTAAAGAGGACTTGGTTGTATGG                                                                                                                                                                                                                                                                                                             <  1:670491/299‑1`  
      `GGTACATGATGAAATCCAAGTAGGCTGCCGTACCGAAGAGATTGGTCAGGTGGTCATTGAGACCGCACAAGAAGCGATGCGCTGGGTTGGAGACCACTGGAACTTCCGGTGTCTTCTGGATACCGAAGGTAAGATGGGTCCTAATTGGGCGATTTGCCACTGATACAGGAGGCTACTCATGAACGAAAGACACTTAACAGGTGCTGCTTCTGAAATGCTAGTAGCCTACAAATTTACCAAAGCTGGGTACACTGTCTATTACCCTATGCTGACTCAGAGTAAAGAGGACTTGGTTGTATGG                                                                                                                                                                                                                                                                                                             <  1:403482/301‑1`  
      `GGTACATGATGAAATCCAAGTAGGCTGCCGTACCGAAGAGATTGCTCAGGTGGTCATTGAGACCGCACAAGAAGCGATGCGCTGGGTTGGAGACCACTGGAACTTCCGGTGTCTTCTGGATACCGAGGGTAAGATGGTTCCTAATTGGGCGATTTGCCACTGATACAGGAGGCTACTCATGAACGAAAGACCCTTACCAGGTGCGGCTTCTGAAATGCTAGTAGCCTACAAATTTACCAAAGCTGGGTACACTGTCTATTACCCTATGCTGACTCAGATTAAAGAGGACTTGGTGGTATGG                                                                                                                                                                                                                                                                                                             >  1:146345/1‑301`  
      `GGTACATGATGAAATCCAAGTAGGATGCCGTACCGAAGAGATTGATCAGGTTGTCATTGAGACCGCACAAGAAGCGATGCGCTTGGTTGGAGACCACTGGATCTCTCGGTGTCTTCTGGATACCGAAGGTAAGATGGGAACTAATTTGGCGATTTTCCACTGATACAGGAGGCTACTCCAGAACGAAAAACACTTAACAGGCGCTGCTTCTGAAAAGCTAGTAGCATAAAAAATTACCAAAGCTGGGTACACTGTCTATTAACATATGCTGACTCAGAGTAAAGAGGACTTGGTTGTATGG                                                                                                                                                                                                                                                                                                             >  1:648143/1‑301`  
         `ACAGGATGAAATCCAAGTAGGCTGCCGTACCGAAGAGATTGCTCAGGTGGTCATTGAGACCGCACAAGAAGCGATGCGCTGGGTTGGAGACCACTGGAACTTCCGGTGTCTTCTGGATACCGAAGGTAAGATGGGTCCTAAGTGGGCGATTTGCCACTGATACAGGAGGCTACTCATGAACGAAAGACACTTAACAGGTGCTGCTTCTGAAATGCTAGTAGCCTACAAATTTACCAAAGCTGGGTACACTGTCTATTACCCTATGCTGACTCAGAGTAAAGAGGACTTGGTTGTATGGAAG                                                                                                                                                                                                                                                                                                          <  1:155061/301‑1`  
          `CATGATGAAATCCAAGTAGGCTGCCGTACCGAAGATATTGCTCAGGTGGTCATTGAGACCGCACAAGAAGCGATGCGCTGGTTTGGAGACCACTGGAACTTCCGGTGTCTTCTGGATACCGAAGGTAAGATGGGTCCTAATTTGGCGATTTGCCACTGATACAGGAGGCTACTCATGAACGAAAGACACTTAACAGGTGCTGCTTCTGAAATGCTAGTAGCCTACAAATTTACCAAAGCTGGGTACACTGTCTATTACCCTATGCTGACTCAGAGTAAAGAGGACTTGGTTGTATGGAAGc                                                                                                                                                                                                                                                                                                         >  1:769537/1‑300`  
              `ATGAAATCCAAGTAGGCTGCCGTACCGAAGAGATTGCTCAGGTGGTCATTGAGACCGCACAAGAAGCGATGCGCTGGGTTGGAGACCACTGGAACTTCCGGTGTCTTCTGGATACCGAAGGTAAGATGGGTCCTAATTGGGCGATTTGCCACTGATACAGGAGGCTACTCATGAACGAAAGACACTTAACAGGTGCTGCTTCTGAAATGCTAGTAGCCTACAAATTTACCAAAGCTGGGTACACTGTCTATTACCCTATGCTGACTCAGAGTAAAGAGGACTTGGTTGTATGGAAGGATTG                                                                                                                                                                                                                                                                                                     >  1:700661/1‑301`  
                `GAAATCCAAGTAGGCTGCCGTACCGAAGAGATTGCTCAGGTGGTCATTGAGACCGCACAAGAAGCGATGCGCTGGGTTGGAGACCACTGGAACTTCCGGTGTCTTCTGGATACCGAAGGTAAGATGGGTCCTAATTGGGCGATTTGCCACTGATACAGGAGGCTACTCATGAACGAAAGACACTTAACAGGTGCTGCTTCTGAAATGCTAGTAGCCTACAAATTTACCAAAGCTGGGTACACTGTCTATTACCCTATGCTGACTCAGAGTAAAGAGGACTTGGTTGTATGGAAGGATTGct                                                                                                                                                                                                                                                                                                   >  1:305059/1‑299`  
                   `ATCTAAGTAGGCTTCCGTACCGAAGAGATTGCTCAGGTGGTCATTGAGACCGCACAAGAAGCGATGCGCTGGGTTGGAGACCACTGGAACTTCCGGTGTCTTCTGGATACCGAAGGTAAGATGGGTCCTAATTGGGCGATTTGCCACTGATACAGGAGACTACTCATGAACGAAAGACACTTAACAGGTGCTGCTTCTGAAATGCTAGTAGCCTACAAATTTACCAAAGCTGGGTACACTGTCTATTACCCTATGCTGACTCAGAGTAAAGAGGACTTGGTTGTATGGAAGGATTGATtct                                                                                                                                                                                                                                                                                                >  1:19999/1‑298`  
                    `TCCAAGTAGGCTGCCGTACCGAAGAGATTGCTCAGGTGGTCATTGAGACCGCACAAGAAGCGATGCGCTGGGTTGGAGACCACTGGAACTTCCGGTGTCTTCTGGATACCGAAGGTAAGATGGGTCCTAATTGGGCGATTTGCCACTGATACAGGAGGCTACTCATGAACGAAAGACACTTAACAGGTGCTGCTTCTGAAATGCTAGTAGCCTACAAATTTACCAAAGCTGGGTACACTGTCTATTACCCTATGCTGACTCAGAGTAAAGAGGACTTGGTTGTATGGAAGGATTGATGACA                                                                                                                                                                                                                                                                                               >  1:627692/1‑301`  
                           `aaagagacagACCGAAGAGATTGCTCAGGTGGTCATTGAGACCGCACAAGAAGCGATGCGCTGGGTTGGAGACCACTGGAACTTCCGGTGTCTTCTGGATACCGAAGGTAAGATGGGTCCTAATTGGGCGATTTGCCACTGATACAGGAGGCTACTCATGAACGAAAGACACTTAACAGGTGCTGCTTCTGAAATGCTAGTAGCCTACAAATTTACCAAAGCTGGGTACACTGTCTATTACCCTATGCTGACTCAGAGTAAAGAGGACTTGGTTGTATGGAAGGATTGATGACATTCGTAG                                                                                                                                                                                                                                                                                        <  1:528437/291‑1`  
                               `TGCCGGACCGAAGAGATGGCTCAGGTGGTCATTGAGACCGCACGAGAAGCGATGCGCTGGGTTGGAGACCACTGGAACTTCCTGTGTCTTCTGGATACCGAAGGTAAGATTGGTCCTAATTGGGCTATTTGCCACTGATACAGGAGGCTACTCATGAACGAAAGACACTTAACAGGTGCTGCTTCTGAAATGCTAGTAGCCTACAAATTTACCAAAGCTGGGTACACTGTCTATTACCCTATGCTGACTCAGAGTAAAGAGGACTTGGTTGTATGGAAGGATTGATGACATTCGTAGTACG                                                                                                                                                                                                                                                                                    <  1:675199/301‑1`  
                                     `ACCGAAGAGATTTCTCAGGTGGTCATTGAGACCGCACAAGAAGCGATGCGCTGGGTTGGAGACCACTGGAACTTCCGGTGTCTTCTGGATACCGAAGGTAAGATGGGTCCTAATTGGGCGATTTGCCACTGATACAGGAGGCTACTCATGAACGAAAGACACTTAACAGGTGCTGCTTCTGAAATGCTAGTAGCCTACAAATTTACCAAAGCTGGGTACACTGTCTATTACCCTATGCTGACTCAGAGTAAAGAGGACTTGGTTGTATGGAAGGATTGATGACATTCGTAGTACGTACATC                                                                                                                                                                                                                                                                              <  1:446546/301‑1`  
                                     `ACCGAAGAGATTGCTCAGGTGGTCATTGAGACCGCACAAGAAGCGATGCGCTGGGTTGGAGACCACTGGAACTTCCGGTGTCTTCTGGATACCGAAGGTAAGATGGGTCCTAATTGGGCGATTTGCCACTGATACAGGAGGCTACTCATGAACGAAAGACACTTAACAGGTGCTGCTTCTGAAATGCTAGTAGCCTACAAATTTACCAAAGCTGGGTACACTGTCTATTACCCTATGCTGACTCAGAGTAAAGAGGACTTGGTTGTATGGAAGGATTGATGACATTCGTAGTACGTACATC                                                                                                                                                                                                                                                                              <  1:381387/301‑1`  
                                      `CCGAAGAGATTGCTCAGGTGGTCATAGAGACCGCACAAGAAGCGATGCGCTGGGTTGGAGACCACTGGAACTTCCGGTGTCTTCTGGATACCGAAGGTAAGATGGGTCCTAATTGGGCGATTTGCCACTGATACAGGAGGCTACTCATGAACGAAAGACACTTAACAGGTGCTGCTTCTGAAATGCTAGTAGCCTACAAATTTACCAAAGCTGGGTACACTGTCTATTACCCTATGCTGACTCAGAGTAAAGAGGACTTGGTTGTATGGAAGGATTGATGACATTCGTAGTACGTACATCA                                                                                                                                                                                                                                                                             <  1:717574/301‑1`  
                                       `ctcgtgggctcggagatgtgtataaGAGACAGCACAAGAAGCGATGCGCTGGGTTGGAGACCACTGGAACTTCCGGTGTCTTCTGGATACCGAAGGTAAGATGGGTCCTAATTGGGCGATTTGCCACTGATACAGGAGGCTACTCATGAACGAAAGACACTTAACAGGTGCTGCTTCTGAAATGCTAGTAGCCTACAAATTTACCAAAGCTGGGTACACTGTCTATTACCCTATGCTGACTCATAGTAAAGAGGACTTGGTTGTATGGAAGGATTGATGACATTCGTAGTACGTACATCAT                                                                                                                                                                                                                                                                            <  1:294611/276‑1`  
                                       `agagacagTTGCTCAGGTGGTCATTGAGACCGCACAAGAAGCGATGCGCTGGGTTGGAGACCACTGGAACTTCCGGTGTCTTCTGGATACCGAAGGTAAGATGGGTCCTAATTGGGCGATTTGCCACTGATACAGGAGGCTACTCATGAACGAAAGACACTTAACAGGTGCTGCTTCTGAAATGCTAGTAGCCTACAAATTTACCAAAGCTGGGTACACTGTCTATTACCCTATGCTGACTCAGAGTAAAGAGGACTTGGTTGTATGGAAGGATTGATGACATTCGTAGTACGTACATCAT                                                                                                                                                                                                                                                                            <  1:540533/293‑1`  
                                       `CGAAGAGATTGCTCAGGTGGTCATTGAGACCGCACAAGAAGCGATGCGCTGGGTTGGATACCACTGGAACTTCCGGTGTCTTCTGGATACCGAAGGTAAGATGGGTCCTAATTGGGCGACTTGCCACTGATACAGGAGGCTACCCATGAACGAAAGACACTTAACAGGTGCTGCTTCTGAAATGCTAGTAGCCTACAAATTTACCAAAGCGGGGTACACTGTCTATTACCCTATGCTGACTCAGAGTAAAGAGGACTTGGTTGTATGGAAGGATTGATGACATTCGTAGTACGTACATCAT                                                                                                                                                                                                                                                                            <  1:390681/301‑1`  
                                                  `CTCAGGTGGTCATTGAGACCGCACAAGAAGCGATGCGCTGGGTTGGAGACCACTGGAACTTCCGGTGTCTTCTGGATACCGAAGGTAAGATGGGTCCTAATTGGGCGATTTGCCACTGATACAGGAAGCTACTCATGAACGAAAGACACTTAACAGGTGCTGCTTCTGAAATGCTAGTAGCCTACAAATTTACCAAAGCTGGGTACACTGTCTATTACCCTATGCTGACTCAGAGTAAAGAGGACTTGGTTGTATGGAAGGATTGATGACATTCGTATTACGTACATCATTTCGTTAGGCC                                                                                                                                                                                                                                                                 >  1:481335/1‑301`  
                                                         `GGTCATTGAGACCGCACAAGAAGCGATGCGCTGGGTTGGAGACCACTGGAACTTCCGGTGTCTTCTGGATACCGAAGGTAAGATGGGTCCTAATTGGGCGATTTGCCACTGATACAGGAGGCTACTCATGAACGAAAGACACTTAACAGGTGCTGCTTCTGAAATGCTAGTAGCCTACAAATTTACCAAAGCTGGGTACACTGTCTATTACCCTATGCTGACTCAGAGTAAAGAGGACTTGGTTGTATGGAAGGATTGATGACATTCGTAGTACGTACATCATTTCGTGAGGCCATTAAGG                                                                                                                                                                                                                                                          <  1:182623/301‑1`  
                                                          `gaaaggGAGACCGAACAAGAAGCGATGCGATGGGTTGGAGACCACTGGAACTTCCGGTGTCTTCTGGATACCGAAGGTAAGATGGGTCCTAATTGGGCGATTTGACACAGATACAGGAGGCTACTCATGAACGAAGGACACTTAACAGGTGCTGCTTCTGAAATGCTAGTAGCCTAAAAATTTACCAAAGCTGGGTACAATGTCTATTACCCTATGCGGACTCAGAGTAAAGAGGACTGGGTTGTATGGAAGGATTGATGACATTCGTAGTACGTACATCATTTCGTGAGGCCATTAAGGA                                                                                                                                                                                                                                                         <  1:397667/295‑1`  
                                                          `GTCATTGAGACCGCACAAGAAGCGATGCGCTGGGTTGGAGACCACTGGAACTTCCGGTGTCTTCTGGATACCGAAGGTAAGATGGGTCCTAATTGGGCGATTTGCCACTGATACAGGAGGCTACTCATGAACGAAAGACACTTAACAGGTGCTGCTTCTGAAATGCTAGTAGCCTACAAATTTACCAAAGCTGGGTACACTGTCTATTACCCTATGCTGACTCAGAGTAAAGAGGACTTGGTTGTATGGAAGGATTGATTACATTCGTAGTACGTACATCATTTCGTGAGGCCATTAAGGA                                                                                                                                                                                                                                                         >  1:52160/1‑301`  
                                                          `GTCATTGAGACCGCACAAGAAGCGATGCGCTGGGTTGGAGACCACTGGAACTTCCGGTGTCTTCTGGATACCGAAGGTAAGATGGGTCCTAATTGGGCGATTTGCCACTGATACAGGAGGCTACTCATGAACGAAAGACACTTAACAGGTGCTGCTTCTGAAATGCTAGTAGCCTACAAATTTACCAAAGCTGGGTACACTGTCTATTACCCTATGCTGACTCAGAGTAAAGAGGACTTGGTTGTATGGAAGGATTGATGACATTCGTAGTACGTACATCATTTCGTGAGGCCATTAAGGA                                                                                                                                                                                                                                                         >  1:170046/1‑301`  
                                                          `GTCATTGAGACCGCACAAGAAGCGATGCGCTGGGTTGGAGACCACTGGAACTTCCGGTGGCTTCTGGATACCGAAGGTAAGATGGGTCCTAATTGGGCGATTTGCCACTGATACAGGAGGCTACTCATGAACGAAAGACACTTAACAGGTGCTGCTTCTGAAATGCTAGTAGCCTACAAATTTACCAAAGCTGGGTACACTGTCTATTACCCTATGCTGACTCAGAGTAAAGAGGACTTGGTTGTATGGAAGGATTGATGACATTCGTAGTACGTACATCATTTCGTGAGGCCATTAAGGA                                                                                                                                                                                                                                                         <  1:55776/301‑1`  
                                                           `TCATGGAGATCGCACAAGAAGCGATGCGCTGGGTTGGAGACCACTGGAACTTCCGGTGTCTTCTGGATACCGAAGGTAAGATGGGTCCTAATTGGGCGATTTGCCACTGATACAGGAGGCTACTCATGAACGAAAGACACTTAACAGGTGCTGCTTCTGAAATGCTAGTAGCCTACAAATTTACCAAAGCTGGGTACACTGTCTATTACCCTATGCTGACTCAGAGTAAAGAGGACTTGGTTGTATGGAAGGATTGATGACATTCGTAGTACGTACATCATTTCGTGAGGCCATTAAGGAC                                                                                                                                                                                                                                                        <  1:135938/301‑1`  
                                                            `CATTGAGACCGCACAAGAAGCGATGCGCTGGGTTGGAGACCACTGGAACTTCCGGTGTCTTCTGGATACCGAAGGTAAGATGGGTCCTAATTGGGCGATTTGCCACTGATACAGGAGGCTACTCATGAACGAAAGACACTTAACAGGTGCTGCTTCTGAAATGCTAGTAGCCTACAAATTTACCAAAGCTGGGTACACTGTCTATTACCCTATGCTGACTCAGAGTAAAGAGGACTTGGTTGTATGGAAGGATTGATGACATTCGTAGTACGTACATCATTTCGTGAGGCCATTACGGACt                                                                                                                                                                                                                                                       >  1:92915/1‑300`  
                                                            `CATTGAGACCGCACAAGAAGCGATGCGCTGGGTTGGAGACCACTGGAACTTCCGGTGTCTTCTGGATACCGAAGGTAAGATGGGTCCTAATTGGGCGATTTGCCACTGATACAGGAGGCTACTCATGAACGAAAGACACTTAACAGGTGCTGCTTCTGAAATGCTAGTAGCCTACAAATTTACCAAAGCTGGGTACACTGTCTATTACCCTATGCTGACCCAGAGTAAAGAGGACTTGGTTGTATGGAAGGATTGATGACATTCGTAGTACGTACATCATTTCGTGAGGCCATTAAGGACA                                                                                                                                                                                                                                                       <  1:618906/301‑1`  
                                                                     `ataagagacAGCGATGGGCTGGGTTGGAGACCAATGGAACTTCCGGTGTATTCTGGATACCGAAGGTAAGATGGGTCCTAATTGGGCGATTTGCCACTGATACAGGAGGCTACTCATGAACGAAAGACACTTAACAGGTGCTGCTTCTGAAATGCTAGTAGCCTACAAATTTACCAAAGCTGGGTACACTGTCTATTACCCTATGCTGACTCAGAGTAAAGAGGACTTGGTTGTATGGAAGGATTGATGACATTCGTAGTACGTACATCATTTCGTGAGGCCATTAAGGACATGCACGAAG                                                                                                                                                                                                                                              <  1:761487/292‑1`  
                                                                            `gtgtataagagaaaaGTTGGAGACCACTGGAACTTCCGGTGTCTTCTGGATACCGAAGGTAAGATGGGTCCTAATTGGGAGATTTGCCACTGATACAGGAGGCTACTCATGAACGAAAGACACTTAACAGGTGCTGCTTCTGAAATGCTAGTAGCCTACAAATTTACCAAAGCTGGGTACACTGTCTATTACCCTATGCTGACTCAGAGTAAAGAGGACTTGGTTGTATGGAAGGATTGATGACATTCGTAGTACGTACATCATTTCGTGAGGCCATTAAGGACATGCACGAAGAGTATGC                                                                                                                                                                                                                                       <  1:725304/286‑1`  
                                                                                `CGATGCGCTGGGTTGGAGACCACTGGAACTTCCGGTGTCTTCTGGATACCGAAGGTAAGATGGGTCCTAATTGGGCGATTTTCCACTGATACAGGAGGCTACTCATGAACGAAAGACACTTAACAGGTGCTGCTTCTGAAATGCTAGTAGCCTACAAATTTACCAAAGCTGGGTACACTGTCTATTACCCTATGCTGACTCAGAGTAAAGAGGACTTGGTTGTATGGAATGATTGATGACATTCGTAGTACGTACATCATTTCGTGAGGCCATTAAGGACATGCACGAAGAGCATGCAGAT                                                                                                                                                                                                                                   <  1:807069/301‑1`  
                                                                                `CGATGCGCTGGGTTGGAGACCACTGGAACTTCCGGTGTCTTCTGGATACCGAAGGTAAGATGGGTCCTAATTGGGCGATTTGCCACTGATACAGGAGGCTACTCATGAACGAAAGACACTTAACAGGTGCTGCTTCTGAAATGCTAGTAGCCTACAAATTTACCAAAGCTGGGTACACTGTCTATTACCCTATGCTGACTCAGAGTAAAGAGGACTTGGTTGTATGGAAGGATTGATGACATTCGTAGTACGTACATCATTTCGTGAGGCCATTAAGGACATGCACGAAGAGTATGCAGAT                                                                                                                                                                                                                                   <  1:61373/301‑1`  
                                                                                    `ggGCTTGGTTGGAGGCCACTGGAACTTCCGGTGTCTTCTGGATACCGAAGGTAAGATGGGTCCCAATTGTGCGATTTTCCACTTTTACAGGAGGCTACTCAGGAACGAAAGACAATTAACAGGTGCTGCTTCTGAAATGCTAGTAGCCCACAAATTTACCAAAGCTGGGTACACTGTCTATTACCCTATGCTGACTCAGAGTAAAGAGGACGTGGTTGTATTGAAGGATTGATGACATTCGTAGTACGTACATCATTTCGTGAGGCCATTAAGGACATGCACGAAGAGTATGCAGATAAGG                                                                                                                                                                                                                               <  1:353954/299‑1`  
                                                                                    `gacagGGGTTGGAGACCACTGGAACTTCCGGTGTCTTCTGGATACCGAAGGTAAGATGGGTCCTAATTGGGCGATTTGCCACTGATACAGGAGGCTACTCATGAACGAAAGACACTTAACAGGTGCTGCTTCTGAAATGCTAGTAGCCTACAAATTTACCAAAGCTGGGTACACTGTCTATTACCCTATGCTGACTCAGAGTAAAGAGGACTTGGTTGTATGGAAGGATTGATGACATTCGTAGTACGTACATCATTTCGTGAGGCCATTAAGGACATGCACGAAGAGTATGCAGATAAGG                                                                                                                                                                                                                               <  1:689614/296‑1`  
                                                                                      `gacaGGTTGGAGACCACTGGAACTTCCGGTGTCTTCTGGATACCGAAGGTAAGATGGGTCCTAATTGGGCGATTTGCCACTGATACAGGAGGCTACTCATGAACGAAAGACACTTAACAGGTGCTGCTTCTGAAATGCTAGTAGCCTACAAATTTACCAAAGCTGGGTACACTGTCTATTACCCTATGCTGACTCAGAGTAAAGAGGACTTGGTTGTATGGAAGGATTGATGACATTCGTAGTACGTACATCATTTCGTGAGGCCATTAAGGACATGCACGAAGAGTATGCAGATAAGGAC                                                                                                                                                                                                                             <  1:20707/297‑1`  
                                                                                      `GCTGGGTTGTAGACCACTGGAACTTCCGGTGTCTTCTGGATACCGAAGGTAAGATGGGTCCTAATTGGGCGATTTGCCACTGATACAGGAGGCTACTCATGAACGAAAGACACTTAACAGGTGCTGCTTCTGAAATGCTAGTAGCCTACAAATTTACCAAAGCTGGGTACACTGTCTATTACCCTATGCTGACTCAGAGTAAAGAGGACTTGGTTGTATGGAAGGATTGATGACATTCGTAGTACGTACATCATTTAGTGAGGCCATTAAGGACATGCACGAAGAGTATGCAGATAAGGAC                                                                                                                                                                                                                             <  1:254047/301‑1`  
                                                                                      `GCTGGGTTGGAGACCACTGGAACTTCCGGTGTCTTCTGGATACCGAAGGTAAGATGGGTCCTAATTGGGCGATTTGCCACTGATACAGGAGGCTACTCATGAACGAAATACACTTAACAGGTGCTGCTTCTGAAATGCTAGTAGCCTACAAATTTACCAAAGCTGGGTACACTGTCTATTAACCTATGCTGACTCAGAGTAAAGAGGACTTGGTTGTATGGAAGGATTGATGACATTCGTAGTACGTACATCATTTCGTGAGGCCATTAAGGACATGCACGAAGAGTATGCAGATAAGGAC                                                                                                                                                                                                                             <  1:453105/301‑1`  
                                                                                         `GGGTTGGAGACCACTGGAACTTCCGGTGTCTTCTGGATACCGAAGGTAAGATGGGTCCTAATTGGGCGATTTGCCACTGATACAGGAGGCTACTCATGAACGAAAGACACTTAACAGGTGCTGCTTCTGAAATGCTAGTAGCCTACAAATTTACCAAAGCTGGGTACACTGTCTATTACCCTATGCTGACTCAGAGTAAAGAGGACTTGGTTGTATGGAAGGATTGATGACATTCGTAGTACGTACATCATTTCGTGAGGCCATTAAGGACATGCACGAAGAGTATGCAGATAAGGACTCT                                                                                                                                                                                                                          >  1:11391/1‑301`  
                                                                                                             `TTCCGTGGTCTTTTGTATACCGCAAGTAAGATGGGTCCTAATTGGGCGATTTGCCACTGATACAGGAAGCTACTCATGAACGAAAGACACTTAACATGTGCTGCTTCTGAAATGCTAGTTGCCTACAAATTTACCAAAGCTGTGTACTCTGTCTATTACCCTATGCTGACTCATAGTAAAGTGTACTTGGTTGTATGGAAGGATTGATGACTTTCGTAGTACGTACATCATTTCTTGTGGCCATTAAGGACATGCACGAAGAGTATGCAGATAAGGACTCTTTCAAACAATCTCCTGCAAC                                                                                                                                                                                                      <  1:586618/301‑1`  
                                                                                                                      `CTTCTGGATACCGAAGGTAAGATGGGTCCTAATTGGGCGATTTGCCACTGATACAGGAGGCTACTCATGAACGAAAGACACTTAACAGGTGCTGCTTCTGAAATGCTAGTAGCCTACAAATTTACCAAAGCTGGGTACACTGTCTATTACCCTATGCTGACTCAGAGTAAAGAGGACTTGGTTGTATGGAAGGATTGATGACATTCGTAGTACGTACATCATTTCGTGAGGCCATTAAGGACATGCACGAAGAGTATGCAGATAAGGACTCTTTCAAACAATCTCCTGCAACAGTACGGGg                                                                                                                                                                                             >  1:570009/1‑300`  
                                                                                                                           `GGATACCGAAGGTAAGATGGGTCCTAATTGGGAGATTTGCCACTGATACAGGAGGCTACTCATGAACGAAAGACACTTAACAGGTGCGGATTCTGAAATGCTAGTAGCCTACAAATTTACCAAAGCT‑GGTACACTGTCTATTAACATATGCTGACTCAGAGTAAAGAGGACTTGGTTGTATGGAAGGATTGATGACATTAGTAGTACGTACATCATTTCGTGAGGCCATTAAGGACATGCACGAAGAGTATGAAGAAAAGGACTATTTCAAACAAACTCATGCAAAAGTACGGatgggagt                                                                                                                                                                                       >  1:268784/1‑293`  
                                                                                                                                `tgtataagagacaGGGTCCTAATTGGGCGATTTGCCACTGATACAGGAGGCTACTCATGAACGAAAGACACTTAACAGGTGCTGCTTCTGAAATGCTAGTAGCCTACAAATTTACCAAAGCTGGGTACACTGTCTATTACCCTATGCTGACTCAGAGTAAAGAGGACTTGGTTGTATGGAAGGATTGATGACATTCGTAGTACGTACATCATTTCGTGAGGCCATTAAGGACATGCACGAAGAGTATGCAGATAAGGACTCTTTCAAACAATCTCCTGCAACAGTACAGGAGGTGTTCTGA                                                                                                                                                                                   <  1:783463/288‑1`  
                                                                                                                                `CCGAAGGTAAGATGGTTCCTAATTGGGCGATTTTCCACTGATACAGGAGGCTACTCATGAACGAAAGACACTTAACAGGTGCTGCTTCTGAAATGCTAGTAGCCTACAAATTTACCAAAGCTGGGTACACTGTCTATTACCCTATGCTGACTCAGAGTAAAGAGGACTTGGTTGTATGGAAGGATTGATGACATTCGTAGTACGTACATCATTTCGTGAGGCCATTAAGGACATGCACGAAGAGTATGCAGATAAGGACTCTTTCAAACAATCTCCTGCAACAGTACGGGAGGTGTTCTGA                                                                                                                                                                                   <  1:683078/301‑1`  
                                                                                                                                  `agagacagGATGGGTCCTAATTGGGCGATTTGCCACTGATACAGGAGGCTACTCATGAACGAAAGACACTTAACAGGTGCTGCTTCTGAAATGCTAGTATCCTACAAATTTACCAAAGCTGGGTACACTGTCTATTACCCTATGCTGACTCAGAGTAAAGAGGACTTGGTTGTATGGAAGGATTGATGACATTCGAAGTACGTACATCATTTCGTGAGGCCATTAAGGACATGCACGAAGAGTATGCAGATAAGGACTCTTTCAAACAATCTCCTGCAACAGTACGGGAGGTGTTCTGATG                                                                                                                                                                                 <  1:419815/293‑1`  
                                                                                                                                  `GAAGGTAAGATGGGTCCTAATTGGGCGATTTGCCACTGATACAGGAGGCTACTCATGAACGAAAGACACTTAACAGGTGCTGCTTCTGAAATGCTAGTAGCCTACAAATTTACCAAAGCTGGGTACACTGTCTATTACCCTATGCTGACTCAGAGTAAAGAGGACTTGGTTGTATGGAAGGATTGATGACATTCGTAGTACGTACATCATTTCGTGAGGCCATTTAGGACATGCACGAAGAGTATGCAGATAAGGACTCTTTCAAACAATCTCCTGCAACAGTACGGGAGGTGTTCTGATG                                                                                                                                                                                 <  1:339320/301‑1`  
                                                                                                                                     `GGTAAGATGGGTCCTAATTGGGCGATTTGCCACTGATACAGTAGGCTACTCATGAACGAAAGACACTTAACAGGTGCTGCTTCTGAAATGCTAGTAGCCTACAAATTTACCAAAGCTGGGTACACTGTCTATTACCCTATGCTGACTCAGAGTAAAGAGGACTTGGTTGTATGGAAGGATTGATGACATTCGTAGTACGTACATCATTTCGTGAGGCCATTAAGGACATGCACGAAGAGTATGCAGATAAGGACTCTTTCAAACAATCTCCTGCAACAGTACGGGAGCTGTctcttataca                                                                                                                                                                              >  1:33297/1‑291`  
                                                                                                                                     `GGTAAGATGGGTCCTAATTGGGCGATTTGCCACTGATACAGGAGGCTACTCATGAACGAAAGACACTTAACAGGTGCTGCTTCTGAAATGCTAGTAGCCTACAAATTTACCAAAGCTGGGTACACTGTCTATTACCCTATGCTGACTCAGAGTAAAGAGGGCTTGGTTGTATGGAAGGATTGATGACCTTCGTAGTACGTACATCATTTCGTGAGGCCATTAAGGACATGCACGAAGAGTATGCAGATAAGGACTCTTTCAAACAATCCCCTGCAACAGTACGGGAGGTGTTCTGATGTCT                                                                                                                                                                              >  1:792465/1‑301`  
                                                                                                                                      `GTAAGATGGGTCCTAATTGGGCGATTTGCCACTGATACAGGAGGCTACTCATGAACGAAAGACACTTAACAGGTGCTGCTTCTGAAATGCTAGTAGCCTACAAATTTACCAAAGCTGGGTACACTGTCTATTACCCTATGCTGACTCAGAGTAAAGAGGACTTGGTTGTATGTAAGGATTGATGACATTCGTAGTACGTACATCATTTCGTGAGGCCATTAAGGACATGCACGAAGAGTATGCAGATAAGGACTCTTTCAAACAATCTCCTTCAACAGTACGGGGAGTGGTCTGATGTCTt                                                                                                                                                                             >  1:164891/1‑300`  
                                                                                                                                                 `CCTAATTGGGCGATTTGCCACTGATACAGGAGGCTACTCATGAACGAAAGACACTTAACAGGTGCTGCTTCTGAAATGCTAGTAGCCTACAAATTTACCAAAGCTGGGTACACTGTCTATTACCCTATGCTGACTCAGAGTAAAGAGGACTTGGTTGTATGGAAGGATTGATGACATTCGTAGTACGTACATCATTTCGTGAGGCCATTAAGGACATGCACGAAGAGTATGCAGATAAGGACTCTTTCAAACAATCTCCTGCAACAGTACGGGctgtctcttatacacatctccgagcccc                                                                                                                                                                  >  1:46659/1‑273`  
                                                                                                                                                   `TAATTGGGCGATTTGCCACTGATACAGGAGGCTACTCATGAACGAAAGACACTTAACAGGTGCTGCTTCTGAAATGCTAGTAGCCTACAAATTTACCAAAGCTGGGTACACTGTCTATTACCCTATGCTGACTCAGAGTAAAGAGGACTTGGTTGTATGGAAGGATTGATGACATTCGTAGTACGTACATCATTTCGTGAGGCCATTAAGGACATGCACGAAGAGTATGCAGATAAGGACTCTTTCAAACAATCTCCTGCAACAGTACGGGAGGTGTTCTGATGTCTGACTACCTGAAAGT                                                                                                                                                                <  1:535591/301‑1`  
                                                                                                                                                                     `gTGATACAGGAGGCTACTCATGAACGAAAGACACTTAACAGGTGCTGCTTCTGAAATGCTAGTAGCCTACAAATTTACCAAAGCTGGGTACACTGTCTATTACCCTATGCTGACTCAGAGTAAAGAGGACTTGGTTGTATGGAAGGATTGATGACATTCGTAGTACGTACATCATTTCGTGAGGCCATTAAGGACATGCACGAAGAGTATGCAGATAAGGACTCTTTCAAACAATCTCCTGCAACAGTACGGGAGGTGTTCTGATGTCTGACTACCTGAAAGTGCTGCAAGCAATCAAAAG                                                                                                                                              <  1:191662/300‑1`  
                                                                                                                                                                                     `CTCATGAACGAAAGACACTTAACAGGTGCTGCTTCTGAAATGCTAGTAGCCTACAAATTTACCAAAGCTGGGTACACTGTCTATTACCCTATGCTGACTCAGAGTAAAGAGGACTTGGTTGTATGGAAGGATTGATGACATTCGTAGTACGTACATCATTTCGTGAGGCCATTAAGGACATGCACGAAGAGTATGCAGATAAGGACTCTTTCAAACAATCTCCTGCAACAGTACGGGAGGTGTTCTGATGTCTGACTACCTGAAAGTGCTGCAAGCAATCAAAAGTTGCCCTAAGACTTTC                                                                                                                              >  1:642965/1‑301`  
                                                                                                                                                                                                 `AGACACTTAACAGGTGCTGCTTCTGAAATGCTAGTAGCCTACAAATTTACCAAAGCTGGGTACACTGTCTATTACCCTATGCTGACTCAGAGTAAAGAGGACTTGGTTGTATGGAAGGATTGATGACATTCGTAGTACGTACATCATTTCGTGAGGCCATTAAGGACATGCACGACGAGTATGCAGATAAGGACTCTTTCAAACAATCTCCTGCAACAGTACGGGAGGTGTTCTGATGTCTGACTACCTGAAAGTGCTGCAAGCAATCAAAAGTTGCCCTAAGACTTTCCAGTCCAACTAT                                                                                                                  >  1:178634/1‑301`  
                                                                                                                                                                                                   `ACACTTAACAGGTGCTGCTTCTGAAATGCTAGTAGCCTACAAATTTACCAAAGCTGGGTACACTGTCTATTACCCTATGCTGACTCAGAGTAAAGAGGACTTGGTTGTATGGAAGGATTGATGACATTCGTAGTACGTACATCATTTCGTGAGGCCATTAAGGACATGCACGACGAGTATGCAGATAAGGACTCTTTCAAACAATCTCCTGCAACAGTACGGGAGGTGTTCTGATGTCTGACTACCTGAAAGTGCTGCAAGCAATCAACAGTTGCCCTAAGACTTTCCAGTCCAACTActt                                                                                                                >  1:309318/1‑298`  
                                                                                                                                                                                                     `ACTTAACAGGTGCTGCTTCTGAAATGCTAGTAGCCTACAAATTTACCAAAGCTGGGTACACTGTCTATTACCCTATGCTGACTCAGAGTAAAGAGGACTTGGTTGTATGGAAGGATTGATGACATTCGTAGTACGTACATCATTTCGTGAGGCCATTAAGGACATGCACGAAGAGTATGCAGATAAGGACTCTTTCAAACAATCTCCTGCAACAGTACGGGAGGTGTTCTGATGTCTGACTACCTGAAAGTGCTGCAAGCAATCAAAAGTTGCCCTAAGACTTTCCAGTCCAACTATGTAC                                                                                                              <  1:593135/301‑1`  
                                                                                                                                                                                                          `ACAGGTGCTGCTTCTGAAATGCTAGTAGCCTACAAATTTACCAAAGCTGGGTACACTGTCTATTACCCTATGCTGACTCAGAGTAAAGAGGACTTGGTTGTATGGAAGGATTGATGACATTCGTAGTACGTACATCATTTCGTGAGGCCATTAAGGACATGCACGAAGAGTATGCAGATAAGGACTCTTTCAAACAATCTCCTGCAACAGTACGGGAGGTGTTCTGATGTCTGACTACCTGAAAGTGCTGCAAGCAATCAAAAGTTGCCCTAAGACTTTCCAGTCCAACTATGTACGGAAC                                                                                                         <  1:425620/301‑1`  
                                                                                                                                                                                                          `ACAGGTGCTGCTTCTGAAATGCTAGTAGCCTACAAATTTACCAAAGCTGGGTACACTGTCTATTACCCTATGCTGACTCAGAGTAAAGAGGACTTGGTTGTATGGAAGGATAGATGACATTCGTAGTACGTACATCATTTCGTGAGGCCATTAAGGACATGCACGAAGAGTATGCAGATAAGGACTCTTTCAAACAATCTCCTGCAACAGTACGGGAGGTGTTCTGATGTCTGACTACCTGAAAGTGCTGCAAGCAATCAAAAGTTGCCCTAAGACTTTCCAGTCCAACTATGTACGGAAC                                                                                                         <  1:12613/301‑1`  
                                                                                                                                                                                                            `AGGTGCTGCTTCTGAAATGCTAGTAGCCTACAAATTTCCCAAAGCTGGGTACACTGTCTATTACCCTATGCTGACTCAGAGTAAAGAGGACTTGGTTGTATGGAAGGATTGATGACATTCGTAGTACGTACATCATTTCGTGAGGCCATTAAGGACATGCACGAAGAGTATGCAGATAAGGACTCTTTCAAACAATCTCCTGCAACAGTACGGGAGGTGTTCTGATGTCTGACTACCTGAAAGTGCTGCAAGCAATCAAAAGTTGCCCTAAGACTTTCCAGTCCAACTATGTACGGAACAA                                                                                                       <  1:499389/301‑1`  
                                                                                                                                                                                                              `GTGCTGCTTCTGAAATGCTAGTAGCCTACAAATTTACCAAAGCTGGGTACACTGTCTATTACCCTATGCTGACTCAGAGTAAAGAGGACTTGGTTGTATGGAAGGATTGATGACATTCGTAGTACGTACATCATTTCGTGAGGCCATTAAGGACATGCACGAAGAGTATGCAGATAAGGACTCTTTCAAACAATCTCCTGCAACAGTACGGGAGGTGTTCTGATGTCTGACTACCTGAAAGTGCTGCAAGCAATCAAAAGTTGCCCTAAGACTTTCCAGTCCAACTATGTACGGAACAATG                                                                                                     >  1:562728/1‑301`  
                                                                                                                                                                                                               `TGCTGCTTCTGAAATGCTAGTAGCCTACAAATTTACCAAAGCTGGGTACACTGTCTATTACCCTATGCTGACTCAGAGTAAAGAGGACTTGGTTGTATGGAAGGATTGATGACATTCGTAGTACGTACATCATTTCGTGAGGCCATTAAGGACATGCACGAAGAGTATGCAGATAAGGACTCTTTCAAACAATCTCCTGCAACAGTACGGGAGGTGTTCTGATGTCTGACTACCTGAAAGTGCTGCAAGCAATCAAAAGTTGCCCTAAGACTTTCCAGTCCAACTATGTACGGAACAATGC                                                                                                    >  1:547693/1‑301`  
                                                                                                                                                                                                                      `TCTGAAATGCTAGTAGCCTACAAATTTACCAAAGCTGGGTACACTGTCTATTACCCTATGCTGACTCAGAGTAAAGAGGACTTGGTTGTATGGAAGGATTGATGACATTCGTAGTACGTACATCATTTCGTGAGGCCATTAAGGACATGCACGAAGAGTATGCAGATAAGGACTCTTTCAAACAATCTCCTGCAACAGTACGGGAGGTGTTCTGATGTCTGACTACCTGAAAGTGCTGCAAGCAATCAAAAGTTGCCCTAAGACTTTCCAGTCCAACTATGTACGGAACAATGCGAGCCTC                                                                                             >  1:631889/1‑301`  
                                                                                                                                                                                                                              `GCTAGTAGCCTACAAATTTACCAAAGCTGGGTACACTGTCTATTACCCTATGCTGACTCAGAGTAAAGAGGACTTGGTTGTATGGAAGGATTGATGACATTCGTAGTACGTACATCATTTCGTGAGGCCATTAAGGACATGCACGAAGAGTATGCAGATAAGGACTCTTTCAAACAATCTCCTGCAACAGTACGGGAGGTGTTCTGATGTCTGACTACCTGAAAGTGCTGCAAGCAATCAAAAGTTGCCCTAAGACTTTCCAGTCCAACTATGTACGGAACAATGCGAGCCTCGTAGCGGg                                                                                     >  1:741228/1‑300`  
                                                                                                                                                                                                                                                    `gtgtataagagacagGTCTATTACCCTATGCTGACTCAGAGTAAAGAGGACTTGGTTGTATGGAAGGATTGATGACATTCGTAGTACGTACATCATTTCGTGAGGCCATTAAGGACATGCACGAAGAGTATGCAGATAAGGACTCTTTCAAACAATCTCCTGCAACAGTACGGGAGGTGTTCTGATGTCTGACTACCTGAAAGTGCTGCAAGCAATCAAAAGTTGCCCTAAGACTTTCCAGTCCAACTATGTACGGAACAATGCGAGCCTCGTAGCGGAGGCCGCTTCCCGTGGTCACATC                                                               <  1:755621/286‑1`  
                                                                                                                                                                                                                                                     `tgtataagagacagGTCTATTACCCTATGCTGACTCAGAGTAAAGAGGACTTGGTTGTATGGAAGGATTGATGACATTCGTAGTACGTACATCATTTCGTGAGGCCATTAAGGACATGCACGAAGAGTATGCAGATAAGGACTCTTTCAAACAATCTCCTGCAACAGTACGGGAGGTGTTCTGATGTCTGACTACCTGAAAGTGCTGCAAGCAATCAAAAGTTGCCCTAAGACTTTCCAGTCCAACTATGTACGGAACAATGCGAGCCTCGTAGCGGAGGCCGCTTCCCGTGGTCACATCT                                                              <  1:230569/287‑1`  
                                                                                                                                                                                                                                                            `GTACACTGTCTATTACCCTATGCTGACTCAGAGTAAAGAGGACTTGGTTGTATGGAAGGATTGATGACATTCGTAGTACGTACATCATTTCGTGAGGCCATTAAGGACATGCACGAAGAGTATGCAGATAAGGACTCTTTCAAACAATCTCCTGCAACAGTACGGGAGGTGTTCTGATGTCTGACTACCTGAAAGTGCTGCAAGCAATCAAAAGTTGCCCTAAGACTTTCCAGTCCAACTATGTACGGAACAATGCGAGCCTCGTAGCGGAGGCCGCTTCCCGTGGTCACATCcctgtctc                                                       >  1:665088/1‑293`  
                                                                                                                                                                                                                                                               `CACTGTCTATTACCCTATGCTGACTCAGAGTAAAGAGGACTTGGTTGTATGGAAGGATTGATGACATTCGTAGTACGTACATCATTTCGTGAGGCCATTAAGGACATGCACGAAGAGTATGCAGATAAGGACTCTTTCAAACAATCTCCTGCAACAGTACGGGAGGTGTTCTGATGTCTGACTACCTGAAAGTGCTGCAAGCAATCAAAAGTTGCCCTAAGACTTTCCAGTCCAACTATGTACGGAACAATGCGAGCCTCGTAGCGGAGGCCGCTTCCCGTGGTCACATCTCGTGCCTGAC                                                    <  1:749523/301‑1`  
                                                                                                                                                                                                                                                                  `TGTCTATTACCCTATGCTGACTCAGAGTAAAGAGGACTTGGTTGTATGGAAGGATTGATGACATTCGTAGTACGTACATCATTTCGTGAGGCCATTAAGGACATGCACGAAGAGTATGCAGATAAGGACTCTTTCAAACAATCTCCTGCAACAGTACGGGAGGTGTTCTGATGTCTGACTACCTGAAAGTGCTGCAAGCAATCAAAAGTTGCCCTAAGACTTTCCAGTCCAACTATGTACGGAACAATGCGAGCCTCGTAGCGGAGGCCGCTTCCCGTGGTCACATCTCGTGCCTGACTAC                                                 <  1:459311/301‑1`  
                                                                                                                                                                                                                                                                   `tTCTATTACCCTATGCTGACTCAGAGTAAAGAGGACTTGGTTGTATGGAAGGATTGATGACATTCGTAGTACGTACATCATTTCGTGAGGCCATTAAGGACATGCACGAAGAGTATGCAGATAAGGACTCTTTCAAACAATCTCCTGCAACAGTACGGGAGGTGTTCTGATGTCTGACTACCTGAAAGTGCTGCAAGCAATCAAAAGTTGCCCTAAGACTTTCCAGTCCAACTATGTACGGAACAATGCGAGCCTCGTAGCGGAGGCCGCTTCCCGTGGTCACATCTCGTGCCTGACTACT                                                <  1:2792/300‑1`  
                                                                                                                                                                                                                                                                   `GTCTATTACCCTATGCTGACTCAGAGTAAAGAGGACTTGGTTGTATGGAAGGATTGATGACATTCGTAGTACGTACATCATTTCGTGAGGCCATTAAGGACATGCACGAAGAGTATGCAGATAAGGACTCTTTCAAACAATCTCCTGCAACAGTACGGGAGGTGTTCTGATGTCTGACTACCTGAAAGTGCTGCAAGCAATCAAAAGTTGCCCTAAGTCTTTCCAGTCCAACTATGTACGGAACAATGCGAGCCTCGTAGCGGAGGCCGCTTCCCGTTGTCACATCTCGTGCCTGACTACT                                                >  1:472990/1‑301`  
                                                                                                                                                                                                                                                                   `GTCTATTACCCTATGCTGACTCAGAGTAAAGAGGACTTGGTTGTATGGAAGGATTGATGACATTCGTAGTACGTACATCATTTCGTGAGGCCATTAAGGACATGCACGAAGAGTATGCAGATAAGGACTCTTTCAAACAATCTCCTGCAACAGTACGGGAGGTGTTCTGATGTCTGACTACCTGAAAGTGCTGCAAGCAATCAAAAGTTGCCCTAAGACTTTCCAGTCCAACTATGTACGGAACAATGCGAGCCTCGTAGCGGAGGCCGCTTCCCGTGGTCACATCTCGTGCCTGACTACT                                                >  1:733979/1‑301`  
                                                                                                                                                                                                                                                                   `GTCTATTACCCTATGCTGACTCAGAGTAAAGAGGACTTGGTTGTATGGAAGGATTGATGACATTCGTAGTACGTACATCATTTCGTGAGGCCATTAAGGACATGCACGAAGAGTATGCAGATAAGGACTCTTTCAAACAATCTCCTGCAACAGTACGGGAGGTGTTCTGATGTCTGACTACCTGAAAGTGCTGCAAGCAATCAAAAGTTGCCCTAAGACTTTACAGTCCAACTATGTACGGAACAATGCGAGCCTCGTAGCGGAGGCCGCTTCCCGTGGTCACATCTCGTGCCTGACTACT                                                >  1:100076/1‑301`  
                                                                                                                                                                                                                                                                     `tTATTACCCTATGCTGACTCAGAGTAAAGAGGACTTGGTTGTATGGAAGGATTGATGACATTCGTAGTACGTACATCATTTCGTGAGGCCATTAAGGACATGCACGAAGAGTATGCAGATAAGGACTCTTTCAAACAATCTCCTGCAACAGTACGGGAGGTGTTCTGATGTCTGACTACCTGAAAGTGCTGCAAGCAATCAAAAGTTGCCCTAAGACTTTCCAGTCCAACTATGTACGGAACAATGCGAGCCTCGTAGCGGAGGCCGCTTCCCGTGGTCACATCTCGTGCCTGACTACTAG                                              <  1:580310/300‑1`  
                                                                                                                                                                                                                                                                     `CTATTACCCTATGCTGACTCAGAGTAAAGAGGACTTGGTTGTATGGAAGGATTGATGAGATTCGTAGTACGTACATCATTTCGTGCGGCCATTAAGGACATGCACGAAGAGTATACAGATAAGGACTCTTTCAAAAAATCTCCTGCCACAGTACGGGAGGTGTTCTGATGTCTGAATACCTGAAAGTGCTGCAAGCAATCAAAAGTTGCCCTAAGACATTCCAGTCCAACTATGTACTGACCAATGCGAGCCTCGTAGCGGAGGCCGCTGACCGTGGTCACATCTCGTGCCTGCCTACTAG                                              >  1:107355/1‑301`  
                                                                                                                                                                                                                                                                     `CTATTACCCTATGCTGACTCAGAGTAAAGAGGACTTGGTTGTATGGAAGGATTGATGACATTCGTAGTACGTACATCATTTCGTGAGGCCATTAAGGACATGCACGAAGAGTATGCAGATAAGGACTCTTTCAAACAATCTCCTGCAACAGTACGGGAAGTGTTCTGATGTCTGACTACCTGAAAGTGCTGCAAGCAATCAAAAGTTGACCTAAGACTTTCCAGTCCAACTATGTACGGAACAATGCGAGCCTCGTAGCGGAGGCCGCTTCCCGTGGTCACATCTCGTGCCTGACTACTAG                                              >  1:410147/1‑301`  
                                                                                                                                                                                                                                                                          `tgtgtataagagacagGAGTAAAGAGGACTTGGTTGTATGGAAGGATTGATGACATTCGTAGTACGTACATCATTTCGTGAGGCCATTAAGGACATGCACGAAGAGTATGCAGATAAGGACTCTTTCAAACAATCTCCTGCAACAGTACGGGAGGTGTTCTGATGTCTGACTACCTGAAAGTGCTGCAAGCAATCAAAAGTTGCCCTAAGACGTTCCAGTCCAACTATGTACGGAACAATGCGAGCCTCGTAGCGGAGGCCGCTTCCCGTGGTCACATCTCGTGCCTGACTACTAGTGGAC                                         <  1:373716/285‑1`  
                                                                                                                                                                                                                                                                          `ACCCTATGCTGACTCAGAGTAAAGAGGACTTGGTTGTATGGAAGGATTGATGACATTCGTAGTACGTACATCATTTCGTGAGGCCATTAAGGACATGCACGAAGAGTATGCAGATAAGGACTCTTTCAAACAATCTCCTGCAACAGTACGGGAGGTGTTCTGATGTCTGACTACCTGAAAGTGCTGCAAGCAATCAAAAGTTGCCCTAAGACTTTCCAGTCCAACTATGTACGGAACAATGCGAGCCTCGTAGCGGAGGCCGCTTCCCGTGGTCACATCTCGTGCCTGACTACTAGTGGAC                                         >  1:775196/1‑301`  
                                                                                                                                                                                                                                                                          `ACCCTATGCTGACTCAGAGTAAAGAGGACTTGGTTGTATGGAAGGATTGATGACATTCGTAGTACGTACATCATTTCGTGAGGCCATTAAGGACATGCACGAAGAGTATGCAGATAAGGACTCTTTCAAACAATCTCCTGCAACAGTACGGGAGGTGTTCTGATGTCTGACTACCTGAAAGTGCTACAAGCAATCAAAAGTTGCCCTAAGACTTTCCAGTCCAACTATGTACGGAACAATGCGAGCCTCGTAGCGGCGGCCGCTTCCCGTGGTCACATCTCGTTCCTGACTACTAGTGGAC                                         >  1:798874/1‑301`  
                                                                                                                                                                                                                                                                          `ACCCTATGCTGACTCAGAGTAAAGAGGACTTGGTTGTATGGAAGGATTGATGACATTCGTAGTACGTACATCATTTCGTGAGGCCATTAAGGACATGCACGAAGAGTATGCAGATAAGGACTATTTCAAACAATCTCCTGCAACAGTACGGGAGGTGTTCTGATGTCTGACTACCTGAAAGTGCTGCAAGCAATCAAAAGTTGCCCTAAGACTTTCCAGTCCAACTATGTACGGAACAATGCGAGCCTCGTAGCGGAGGCCGCTTCCCGTGGTCACATCTCGTGCCTGACTACTAGTGGAC                                         <  1:824111/301‑1`  
                                                                                                                                                                                                                                                                               `ATGCTGACTCAGAGTAAATAGGACTTGGTTGTATGGAAGGATTGATGACATTCGTAGTACGTACATCATTTCGTGAGGCCATTAAGGACATGCACGAAGAGTATGCAGATAAGGACTCTTTCAAACAATCTCCTGCAACAGTACGGGAGGTGTTCTGATGTCTGACTACCTGAAAGTGCTGCAAGCAATCAAAAGTTGCCCTAAGACTTTCCAGTCCAACTATGTACGGAACAATGCGAGCCTCGTAGCGGAGGCCGCTTCCCGTGGTCACATCTCGTGCCTGACTACTAGTGctgtctct                                    >  1:506134/1‑293`  
                                                                                                                                                                                                                                                                               `ATGCTGACTCAGAGTAAAGAGGACTTGGTTGTATGGAAGGATTGATGACATTCGTAGTACGTACATCATTTCGTGAGGCCATTAAGGACATGCACGAAGAGTATGCAGATAAGGACTCTTTCAAACAATCTCCTGCAACAGTACGGGAGGTGTTCTGATGTCTGACTACCTGAAAGTGCTGCAAGCAATCAAAAGTTTCCCTAAGACTTTCCAGTCCAACTATGTACGGAACAATGCGAGCCTCGTAGCGGAGGCCGCTTCCCGTGGTCACATCTCGTGCCTGACTACTAGTGGACGTAAC                                    >  1:320365/1‑301`  
                                                                                                                                                                                                                                                                                 `GCTGACTCAGAGTAAAGAGGACTTGGTTGTATGGAAGGATTGATGACATTCGTAGTACGTACATCATTTCGTGAGGCCATTAAGGACATGCACGAAGAGTATGCAGATAAGGACTCTTTCAAACAATCTCCTGCAACAGTACGGGAGGTGTTCTGATGTCTGACTACCTGAAAGTGCTGCAAGCAATCAAAAGTTGCCCTAAGACTTTCCAGTCCAACTATGTACGGAACAATGCGAGCCTCGTAGCGGAGGCCGCTTCCCGTGGTCACATCTCGTGCCTGACTACTAGTGGACctgtctc                                  >  1:432353/1‑294`  
                                                                                                                                                                                                                                                                                          `GAGTAAAGAGGACTTGGTTGTATGGAAGGATTGATGACATTCGTAGTACGTACATCATTTCGTGAGGCCATTAAGGACATGCACGAAGAGTATGCAGATAAGGACTCTTTCAAACAATCTCCTGCAACAGTACGGGAGGTGTTCTGATGTCTGACTACCTGAAAGTGCTGCAAGCAATCAAAAGTTGCCCTAAGACTTTCCAGTCCAACTATGTACGGAACAATGCGAGCCTCGTAGCGGAGGCCGCTTCCCGTGGTCACATCTCGTGCCTGACTACTAGTGGACGTAACGGTGGCGtctg                         >  1:241166/1‑297`  
                                                                                                                                                                                                                                                                                                   `ttataagggacagTGGAAGGATTGATGACATTCGTAGTACGTACATCATTTCGTGAGGCCATTAAGGACATGCACGAAGAGTATGCAGATAAGGACTCTTTCAAACAATCTCCTGCAACAGTACGGGAGGTGTTCTGATGTCTGACTACCTGAAAGTGCTGCAAGCAATCAAAAGTTGCCCTAAGACTTTCCAGTCCAACTATGTACGGAACAATGCGAGCCTCGTAGCGGAGGCCGCTTCCCGTGGTCACATCTCGTGCCTGACTACTAGTGGACGTAACGGTGGCGCTTGGGAAATCAC                <  1:815406/288‑1`  
                                                                                                                                                                                                                                                                                                      `aaacatgggATGGAAGGATTGATGACAGTCGTAGTACGTACATCATTTCGTGAGGCCATTAAGGACATGCACGAAGAGTATGCAGATAAGGACTCTTTCAAACAATCTCATGAAACAGTACGGGAGGTGTTCTGATGTCTGACTACCTTAAAGTGCGGCAAGCAATCAAAAGTTGCCCTAAGACTTTCCAGTCCAACTATGTACGGAACAATGCGAGCCTCGTAGCGGAGGCCGCTTCCCGTGGTCACATCTCGTGCTTGACTACTAGTGGACGTAACGGTGGCGCTTGGGAAATCACTGC             <  1:262584/292‑1`  
                                                                                                                                                                                                                                                                                                      `CTTGGTTGTATGGAAGGATTGATGACATTCGTAGTACGTACATCATTTCGTGAGGCCATTAAGGACATGCACGAAGAGTATGCAGATAAGGACTCATTCAAACAATCTCCTGCAACAGTACGGGAGGTGTTCTGATGTCTGACTACCTGAAAGTGCTGCAAGCAATCAAAAGTTGCCCTAAGACTTTCCAGTCCAACTATGTACGGAACAATGCGAGCCTCGTAGCGGAGGCCGCTTCCCGTGGTCACATCTCGTGCCTGACTACTAGTGGACGTAACGGTGGCGCTTGGGAAATCACTGC             <  1:70985/301‑1`  
                                                                                                                                                                                                                                                                                                         `GGTTGTATGGAAGGATTGATGACATTCGTAGTACGTACATCATTTCGGGAGGCCATTAAGGACATGCACGAAGAGTATGCAGATAAGGACTCTTTCAAACAATCTCCTGCAACAGTACGGGAGGTGTTCTGATGTCTGACTACCTGAAAGTGCTGCAAGCAATCAAAAGTTGCCCTAAGACTTTCCAGTCCAACTATGTACGGAACAATGCGAGCCTCGTAGAGGAGGCCGCTTCCCGTGGTCACATCTCGTGCCTGACTACTAGTGGACGTAACGGTGGCGCTTGGGAAATCACTGCTTC          <  1:221255/301‑1`  
                                                                                                                                                                                                                                                                                                             `GTATGGAAGGATTTATGACATTTGTAGTACGTACATCATTTCGTGATGCCATTAAGGACATGCACGAAGAGTATGCAGATAAGGACTCTTTCAAACAATCTCCTGCAACAGTACGGGAGGTGTTCTGATGTCTGACTACCTGAAAGTGCTGCAAGCAATCAAAAGTTGCCCTAAGACTTTCCAGTCCAACTATGTACGGAACAATGCGAGCCTCGTAGCGGAGGCCGCTTCCCGTGGTCACATCTCGTGCCGGACTACGAGAGGACGTAAAGGTGGCGCTTGGGAAAGCACTGCTTCCGGg      >  1:656468/1‑300`  
                                                                                                                                                                                                                                                                                                             `GTATGGAAGGATTGATGACATTCGTAGTACGTACATCATTTCGTGAGGCCATTAAGGACATGCACGAAGAGTATGCAGATAAGGACTCTTTCAAACAATCTCCTGCAACAGTACGGGAGGTGTTCTGATGTCTGACTCCCTGAAAGTGCTGCAAGCAATCAAAAGTTGCCCTAAGACTTTCCAGTCCAACTATGTACGGAACAATGCGAGCCTCGTAGCGGAGGCCGCTTCCCGTGGTCACATCTCGTGCCTGACTACTAGTGGACGTAACGGTGGCGCTTGGGAAATCACTGCTTCCGGT      >  1:346889/1‑301`  
                                                                                                                                                                                                                                                                                                             `GTATGGAAGGATTGATGACATTCGTAGTACGTACATCATTTCGTGAGGCCATTAAGGACATGCACGAAGAGTATGCAGATAAGGACTCTTTCAAACAATCTCCTGCAACAGTACGGGAGGTGTTCTGATGTCTGACTACCTGAAAGTGCTGCAAGCAATCAAAAGTTGCCCTAAGACTTTCCAGTCCAACTATGTACGGAACAATGCGAGCCTCGTAGCGGAGGCCGCTTCCCGTGGTCACATCTCGTGCCTGACTACTAGTGGACGTAACGGTGGCGCTTGGGAAATCACTGCTTCCGGT      >  1:443011/1‑301`  
                                                                                                                                                                                                                                                                                                             `GTATGGAAGGATTGATGACATTCGTAGTACGTACATCATTTCGTGAGGCCATTAAGGACATGCACGAAGAGTATGCAGATAAGGACTCTTTCAAACAATCTCCTGCAACAGTACGGGAGGTGTTCTGATGTCTGACTACCTGAAAGTGCTGCAAGCAATCAAAAGTTGCCCTAAGACTTTCCAGTCCAACTATGTACGGAACAATGCGAGCCTCGTAGCGGAGGCCGCTTCCCGTGGTCACATCTCGTGCCTGACTACTAGTGGACGTAACGGTGGCGCTTGGGAAATCACTGCTTCCGGT      <  1:512423/301‑1`  
                                                                                                                                                                                                                                                                                                             `GTATGGAAGGATTGATGACATTCGTAGTACGTACATCATTTCGTGAGGCCATTAAGGACATGCACGAAGAGTATGCAGATAAGGACTCTTTCAAACAATCTCCTGCAACAGTACGGGAGGTGTTCTGATGTCTGACTACCTGAAAGTGCTGCAAGCAATCAAAAGTTGCCCTAAGACTTTCCAGTCCAACTATGTACGGAACAATGCGAGCCTCGTAGCGGAGGCCGCTTCCCGTGGTCACATCTCGTGCCTGACTACTAGTGGACGTAACGGTGGCGATTGGGActgtctcttatacata      >  1:575123/1‑285`  
                                                                                                                                                                                                                                                                                                               `ATGGAAGGATTGATGACATTCGTAGTACGTACATCATTTCGTGAGGCCATTAAGGACATGCACGAAGAGTATGCAGATAAGGACTCTTTCAAACAATCTCCTGCAACAGTACGGGAGGTGTTCTGATGTCTGACTACCTGAAAGTGCTGCAAGCAATCAAAAGTTGCCCTAAGACTTTCCAGTCCAACTATGTACGGAACAATGCGAGCCTCGTAGCGGAGGCCGCTTCCCGTGGTCACATCTCGTGCCTGACTACTAGTGGACGTAACGGTGGCGCTTGGGAAATCACTGCTTCCGGTAC    >  1:83718/1‑301`  
                                                                                                                                                                                                                                                                                                               `ATGGAAGGATTGATGACATTCGTAGTACGTACATCATTTCGTGAGGCCATTAAGGACATGCACGAAGAGTATGCAGATAAGGACTCTTTCAAACAATCTCCTGCAACAGTACGGGAGGTGTTCTGATGTCTGACTACCTGAAAGTGCTGCAAGCAATCAAAAGTTGCCCTAAGACTTTCCAGTCCAACTATGTACGGAACAATGCGAGCCTCGTAGCGGAGGCCGCTTCCCGTGGTCACATCTCGTGCCTGACTACTAGTGGACGTAACGGTGGCGCTTGGGAAATCACTGCTTCCGGTAC    >  1:120215/1‑301`  

                                                                                                                                                                                                                                                                                                                                                                                                                                                                                                                                                                                                                                
`GGCATGGGTACATGATGAAATCCAAGTAGGCTGCCGTACCGAAGAGATTGCTCAGGTGGTCATTGAGACCGCACAAGAAGCGATGCGCTGGGTTGGAGACCACTGGAACTTCCGGTGTCTTCTGGATACCGAAGGTAAGATGGGTCCTAATTGGGCGATTTGCCACTGATACAGGAGGCTACTCATGAACGAAAGACACTTAACAGGTGCTGCTTCTGAAATGCTAGTAGCCTACAAATTTACCAAAGCTGGGTACACTGTCTATTACCCTATGCTGACTCAGAGTAAAGAGGACTTGGTTGTATG‑‑‑‑‑‑‑‑‑‑‑‑‑‑‑‑‑‑‑‑‑‑‑‑‑‑‑‑‑‑‑‑‑‑‑‑‑‑‑‑‑‑‑‑‑‑‑‑‑‑‑‑‑‑‑‑‑‑‑‑‑‑‑‑‑‑‑‑‑‑‑‑‑‑‑‑‑‑‑‑‑‑‑‑‑‑‑‑‑‑‑‑‑‑‑‑‑‑‑‑‑‑‑‑‑‑‑‑‑‑‑‑‑‑‑‑‑‑‑‑‑‑‑‑‑‑‑‑‑‑‑‑‑‑‑‑‑‑‑‑‑‑‑‑‑‑‑‑‑‑‑‑‑‑‑‑‑‑‑‑‑‑‑‑‑‑‑‑‑‑‑‑‑‑‑‑‑‑‑‑‑‑‑‑‑‑‑‑‑‑‑‑‑‑‑‑‑‑‑‑‑‑‑‑‑‑‑‑‑‑‑‑‑‑‑‑‑‑‑‑‑‑‑‑‑‑‑‑‑‑‑‑‑‑‑‑‑‑‑‑‑‑‑‑‑‑‑‑‑‑‑‑‑‑‑‑‑‑‑‑‑‑‑‑‑‑‑‑‑‑‑‑‑‑‑‑‑‑‑‑‑‑‑‑‑‑‑‑‑‑‑‑‑‑‑‑‑‑‑‑  >  NC_001604.1/16299‑16604`  
`‑‑‑‑‑‑‑‑‑‑‑‑‑‑‑‑‑‑‑‑‑‑‑‑‑‑‑‑‑‑‑‑‑‑‑‑‑‑‑‑‑‑‑‑‑‑‑‑‑‑‑‑‑‑‑‑‑‑‑‑‑‑‑‑‑‑‑‑‑‑‑‑‑‑‑‑‑‑‑‑‑‑‑‑‑‑‑‑‑‑‑‑‑‑‑‑‑‑‑‑‑‑‑‑‑‑‑‑‑‑‑‑‑‑‑‑‑‑‑‑‑‑‑‑‑‑‑‑‑‑‑‑‑‑‑‑‑‑‑‑‑‑‑‑‑‑‑‑‑‑‑‑‑‑‑‑‑‑‑‑‑‑‑‑‑‑‑‑‑‑‑‑‑‑‑‑‑‑‑‑‑‑‑‑‑‑‑‑‑‑‑‑‑‑‑‑‑‑‑‑‑‑‑‑‑‑‑‑‑‑‑‑‑‑‑‑‑‑‑‑‑‑‑‑‑‑‑‑‑‑‑‑‑‑‑‑‑‑‑‑‑‑‑‑‑‑‑‑‑‑‑‑‑‑‑‑‑‑‑‑‑‑‑‑‑‑‑‑‑‑‑‑‑‑‑‑‑‑‑‑‑‑‑‑‑‑‑‑‑‑‑‑‑‑‑‑‑‑‑‑‑gtatgGAAGGATTGATGACATTCGTAGTACGTACATCATTTCGTGAGGCCATTAAGGACATGCACGAAGAGTATGCAGATAAGGACTCTTTCAAACAATCTCCTGCAACAGTACGGGAGGTGTTCTGATGTCTGACTACCTGAAAGTGCTGCAAGCAATCAAAAGTTGCCCTAAGACTTTCCAGTCCAACTATGTACGGAACAATGCGAGCCTCGTAGCGGAGGCCGCTTCCCGTGGTCACATCTCGTGCCTGACTACTAGTGGACGTAACGGTGGCGCTTGGGAAATCACTGCTTCCGGTACTC  >  NC_001604.1/17028‑17327`

  

Alignment Legend

Aligned base mismatch/match (shaded by quality score): `ATCG/ATCG` `< 3 ≤` `ATCG/ATCG` `< 14 ≤` `ATCG/ATCG` `< 27 ≤` `ATCG/ATCG` `< 37 ≤` `ATCG/ATCG` `< 38 ≤` `ATCG/ATCG`

Unaligned base: `atcg`    Masked matching base: `atcg`    Alignment gap: `‑`    Deleted base: `‑`

Reads not counted as support for junction

read\_name Not counted due to insufficient overlap past the breakpoint.

read\_name Not counted due to not crossing MOB target site duplication.
